# Modelling Covid-19 under uncertainty: what can we expect?

**DOI:** 10.1007/s10198-020-01202-y

**Published:** 2020-06-03

**Authors:** Meimei Wang, Steffen Flessa

**Affiliations:** University of Greifswald, Friedrich-Loeffler-Straße 70, 17487 Greifswald, Germany

**Keywords:** I100

The Covid-19 pandemic constitutes one of the greatest medical, social, economic and political challenges for the last decades. Millions have been infected, hundred thousands have died and the economic loss reaches totals that are gigantic even for macro economists used to big numbers. Within a short period of time an enormous number of far-reaching decisions have to be made within a highly complex system of interdependent virological, pharmaceutical, behavioural, legal, social and economic frameworks. Hardly ever before we have experienced such a strong set of policies based on so little evidence as in the last few months since Sars-Cov-2 became a life-threatening challenge for the health systems and the population of this world [1, 2].

Covid-19 affects all subsystems and it is obvious that fighting this disease goes far beyond the health care arena. This high complexity and dynamics calls for reliable decision-bases. In particular, health decision-makers have to know the epidemiologic and economic consequences of interventions. As wrong decisions might result in loss of life, chaotic over-burdening of health care services, unemployment and even social unrest affecting millions, evidence-based policy making would be a must—but there is no evidence about forecasting the future.

**Fig. 1:**
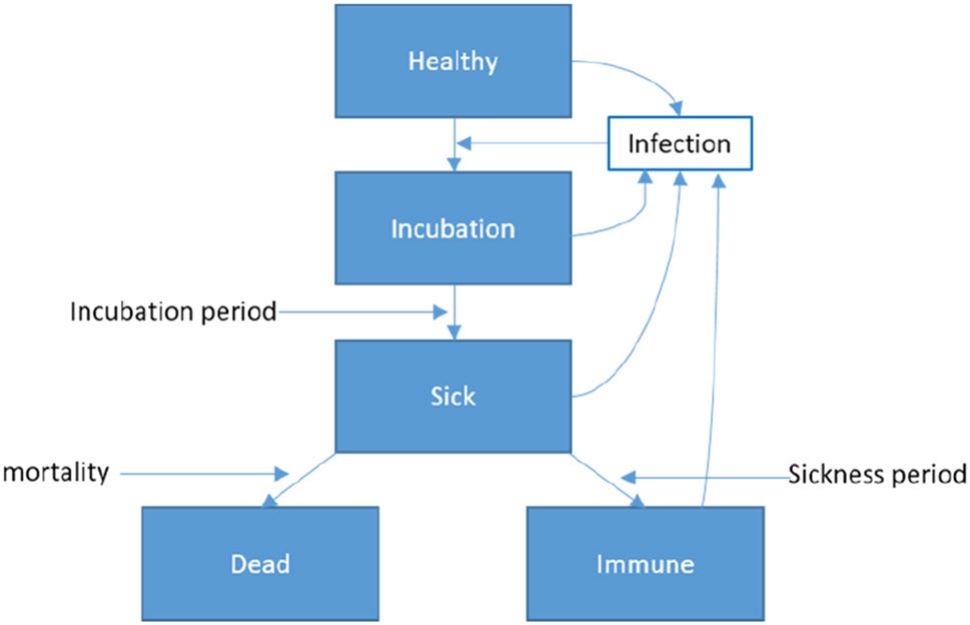
Covid-19 model structure

What can scientists and in particular health economists contribute to this struggle for evidence? Seeing the high complexity and dynamics of the Covid-19 system, modelling becomes a must to support health-policy decisions [3]. Already very early, models predicting the spread of this disease were published. Markov and System Dynamics models usually divide the population into compartments of being susceptible to the disease (S), actively infected with the disease (I), and recovered (or dead) and no longer contagious (R), i.e., they use a SIR-model structure [4–7] (Fig. 1). As Covid-19 is an infectious disease, the likelihood of falling sick depends on the share of the infectious population in the total population, i.e., the infection risks changes with every person who is infected, falls sick, dies or recovers. Consequently, homogenous Markov chains where the transition probabilities are constant are inadequate for modelling infectious diseases. Inhomogeneous Markov chains [8] where the transition probabilities are re-calculated for every time interval are identical with System Dynamics Models [7, 9] if the time intervals are sufficiently frequent.

Common models used to forecast infectious diseases are (system) dynamics and discrete event simulations [10]. The system dynamics model [11–13] defines difference equations for the healthy, infected, sick and immune population, e.g.

$$H_{t + 1} = H_{t} - \frac{{H_{t} }}{{H_{t} + I_{t} + S_{t} + M_{t} }} \cdot \frac{{I_{t} + S_{t} }}{{H_{t} + I_{t} + S_{t} + M_{t} }} \cdot H_{t} \cdot r$$

$$I_{t + 1} = I_{t} + \frac{{H_{t} }}{{H_{t} + I_{t} + S_{t} + M_{t} }} \cdot \frac{{I_{t} + S_{t} }}{{H_{t} + I_{t} + S_{t} + M_{t} }} \cdot H_{t} \cdot r - \frac{{I_{t} }}{i}$$

$$S_{t + 1} = S_{t} + \frac{{I_{t} }}{i} - S_{t} \cdot m - \frac{{S_{t} }}{s}$$

$$T_{t + 1} = T_{t} + S_{t} \cdot m$$

$$M_{t + 1} = M_{t} + \frac{{S_{t} }}{s}$$

With

**Table:** 

Variables	Description
*H*_*t*_	Healthy in *t*
*I*_*t*_	Incubating in *t*
*S*_*t*_	Sick in *t*
*M*_*t*_	Immune in *t*
*T*_*t*_	Death cases in *t*

**Table:** 

Constants		Value	Source
*H*_0_	Healthy in *t* = 0	1,000,000	
*m*	Mortality rate	0.05	[14]
*s*	Sickness period	14 d	[15]
*i*	Incubation period	5 d	[15]
*R*_0_	Basic reproductive rate	3	[16]
*r*	Infection rate per day: *r* = *R*_0_/(*i* + *s*)	0.158	

**Fig. 2:**
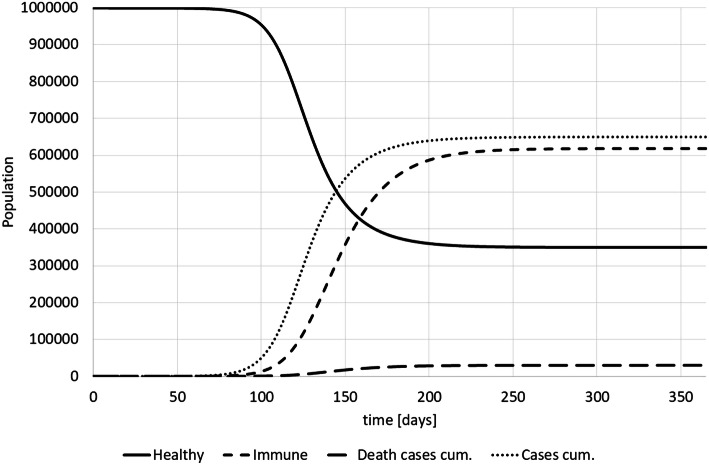
Covid-19 model SIR-results (own)

Based on this model we can calculate the spread of the disease and simulate the impact of interventions (see Fig. 2).

System Dynamics Models can be much more complex, i.e., they can distinguish between different population groups (e.g. age-sets, general health status of population) and include non-infectious periods or different levels of health care. However, all System Dynamics Models are compartment models assuming that a population within one compartment is homogenous. In reality, people show different behaviour and social interactions which is in particular relevant for the spread of a contagious disease. Consequently, in particular the first weeks of a disease or an intervention cannot be properly analysed with system dynamics models. Instead, simulation models with individuals (discrete event simulation, DES) [17, 18] and with individual characteristics (agent-based simulation, ABS) [19, 20] are used to forecast the (early) spread of a disease. Here individuals with pre-defined behaviour interact and infections of individuals are computed. If the SIR-model is sufficiently complex and a higher number of individuals exists in each compartment, SIR-models and ABS will lead the similar results while early stages of a disease outbreak or of an intervention will be more correct with ABS [21, 22].

The formulation and computation of even the best Covid-19 models are under severe uncertainty [23]. Firstly, there is uncertainty considering biological and medical facts, i.e., most medical parameters are unknown or strongly uncertain. For instance, we do not know exactly the relevance of the viral load, the determinants of the case fatality rates (e.g., chronic-degenerative diseases, obesity), the influence of earlier infections with other Corona viruses or the general immunity (e.g., caused by a BCG-vaccination). This uncertainty challenges modelling and policy advice. In addition, some parameters might change in the course of the disease due to mutation of the virus.

Secondly, the vagueness of medical care structures and data is another source of uncertainty in disease modelling. For instance, the situation in French and Italian hospitals during the most severe Covid-19 crises in March 2020 did definitely not follow official guidelines of intensive care. Until today there is no standard of treatment of patients affected by Sars-Cov-2 which makes modelling difficult. Thirdly, many economic facts are unknown. Currently, we can only guess what a vaccination or anti-Corona drug will cost. And the total burden of Covid-19 cannot really be assessed fully. Finally, health-policy support for Covid-19 requires some knowledge of social patterns, such as contact behaviour. We can estimate the basic reproductive rate but we do not have a model to predict the impact of wearing masks, home–office, closing schools etc. It will take years until we understand the complexity of the spread of Sars-Cov-2 in sufficient details.

**Fig. 3:**
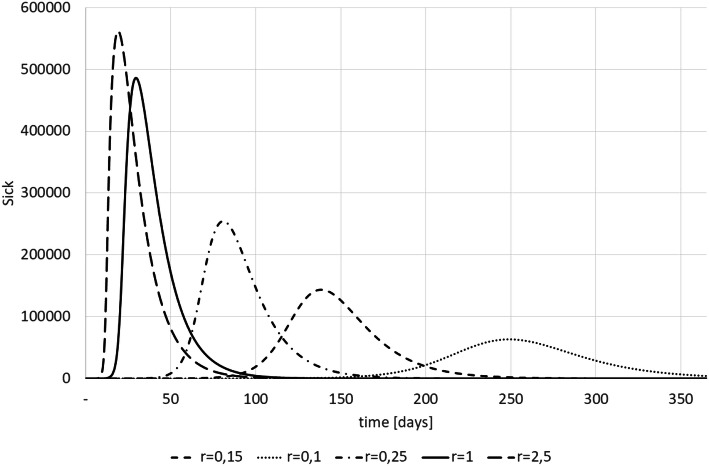
Sick patients and infection rate (results from own simulations)

The impact of a structure or parameter error on the simulation results (and thus on the policy advise) can be tremendous. For instance, Fig. 3 shows the consequences of differing infection rates (*r*). This statistics is the probability of getting infected per day if a non-infected encounters an infected, i.e., *r* = *R*_0_/(*i* + *s*). It is obvious that this parameter is of utmost relevance for the analysis whether the health systems capacity suffices—but it depends on many behaviour variables and is not stable over time (e.g., seasonal influences).

Does that mean that health economists should stop developing models and wait until virologists and others have determined all necessary parameters? This would be irresponsible as modelling is absolutely essential to support health-policy making today. They have to make decisions today and we have to support them with evidence. Instead, we have to invest effort to deal with uncertainty in our models [24, 25]. The following strategies are recommendable and should be a standard for Covid-19 modelling [23].

- *Thorough literature analysis* The existing literature (including existing models) should be properly analysed to determine structures and parameters.
- *Calibration* The model must be calibrated until it fits existing data as closely as possible, i.e. until the simulated results are close to the real data [26].
- *Sensitivity analyses* Parameters must be altered and consequences recorded [27] to develop a measure how reliable the results are as basis for decision-making.
- *Scenarios* indicate the consequences of structural changes and parameter changes.
- *Caution* All results must be interpreted with great caution and humbleness. The scenarios will produce intervals of incidence and prevalence rates as well as bands of cost-effectiveness that are the basis for political decisions. It is unlikely that the models produce the only one and right answer, but they will provide likelihoods.

Modelling Covid-19 only a few months after its break-out and under severe uncertainty will not produce all-and-forever true figures. Instead, we should use these strategic models as “modelling for insights, not for numbers” [28]. They can serve the scientists and politicians to understand the system much better, to point at research gaps and to give some principle answers to urgent questions. These answers cannot be precise figures, but our modelling results will permit humble and cautious policy advice.
